# Clinical predictive value of *in vitro* anticancer drug sensitivity test for the therapeutic effect of adjuvant chemotherapy in patients with stage II–III colorectal cancer

**DOI:** 10.3892/mco.2013.102

**Published:** 2013-04-11

**Authors:** EIJI MEKATA, HIROMICHI SONODA, TOMOHARU SHIMIZU, TAKESHI TATSUTA, TOMOHIRO YAMAGUCHI, YOSHIHIRO ENDO, TOHRU TANI

**Affiliations:** Department of Surgery, Shiga University of Medical Science, Otsu, Shiga 520-2192, Japan

**Keywords:** collagen gel droplet-embedded culture drug sensitivity test, colorectal cancer, adjuvant chemotherapy

## Abstract

Clinically useful predictors of the efficacy of adjuvant chemotherapy following curative colorectal surgery remain to be determined. In the present study, we investigated the clinical utility of the collagen gel droplet-embedded culture drug sensitivity test (CD-DST) as a predictor of the therapeutic response to 5-fluorouracil (5-FU)-based adjuvant chemo-therapy in patients with stage II–III colorectal cancer. CD-DST was conducted using tumor samples surgically obtained from 189 patients. The therapeutic effect of 5-FU-based regimens between high (high-group) and low (low-group) sensitivity groups and a group that did not receive chemotherapy [CTx(-) group] was compared. CD-DST was successfully performed in 151 out of the 189 patients (79.9%), 87 of whom received 5-FU-based adjuvant chemotherapy after surgery. Twenty-seven of these 87 patients (31.0%) were classified as the high-group and the remaining 60 (69.0%) as the low-group. The 5-year recurrence-free survival (RFS) in the high-group was significantly higher compared to that in the low- and the CTx(-) groups. No differences in the 5-year RFS were observed between the low- and CTx(-) groups. In conclusion, CD-DST appears to be useful for predicting the therapeutic response to 5-FU-based adjuvant chemotherapy in patients with stage II-III colorectal cancer.

## Introduction

In western countries, l-oxaliplatin (l-OHP)-based adjuvant chemotherapy remains the standard adjuvant chemotherapy for stage III colorectal cancer (1,2). Furthermore, 5-fluorouracil (5-FU)-based adjuvant chemotherapy is one of the standard adjuvant chemotherapy choices for stage III colorectal cancer in Japan (3). Predictive markers for adjuvant chemotherapy have also been evaluated, with several molecular predictors being considered as satisfactory predictive markers for the efficacy of 5-FU-based chemotherapy (4–6) and 5-FU/l-OHP combination chemotherapy (7). However, clinically useful predictors that determine whether patients should be administered 5-FU-based or l-OHP-based chemotherapy remain to be identified. Moreover, whether adjuvant chemotherapy is required for patients with stage II colorectal cancer has not yet been determined.

The collagen gel droplet-embedded culture drug sensitivity test (CD-DST) is a new *in vitro* anticancer drug sensitivity test. One of the advantages of CD-DST, compared to previous anticancer drug sensitivity tests, is that it favors the growth of tumor tissues in long-term cultures, due to its use of a three-dimensional growth assay with an image analysis device that is able to differentiate between cancer cells and fibroblasts (8). Previous studies reported that CD-DST may be useful when devising optimal treatment strategies for ovarian (9) or gastrointestinal cancer (10–12). However, the clinical utility of CD-DST in the prediction of the response to adjuvant chemotherapy for colorectal cancer remains to be determined. Therefore, we attempted to investigate the clinical utility of CD-DST as a predictor of the therapeutic response to 5-FU-based adjuvant chemotherapy in patients with stage II–III colorectal cancer.

## Patients and methods

### Patient characteristics

This study included 246 patients with stage II–III colorectal cancer who underwent curative surgery between January, 2000 and December, 2007 at the Department of Surgery, Shiga University of Medical Science (Otsu, Japan).

A total of 108 men and 138 women with a median age at the time of surgery of 66.06 years (range, 35–92 years) were included in this study. As regards tumor location, 174 patients had colon and 72 had rectal cancer. Stage II cancer was diagnosed in 119 patients (119/246, 48.4%) and stage III disease in 127 patients (127/246, 51.6%). Staging was based on the general rules for clinical and pathological studies on cancer of the colon, rectum and anus (13).

Generally, stage III patients were younger compared to stage II patients (P=0.025). In the stage II group, the number of colon cancer patients was significantly higher compared to that of rectal cancer patients (P=0.0014). No differences in gender and histological type were observed between stage II and III patients.

The number of stage III patients who received adjuvant chemotherapy was significantly higher compared to that of stage II patients (P=0.0015).

### Study protocol

**Figure 1.**
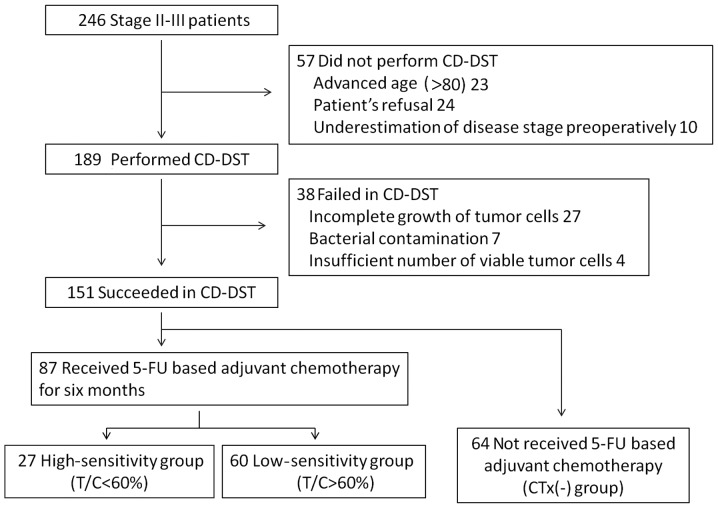
Study protocol. T, total colony volume of treated cells; C, total colony volume of untreated cells; 5-FU, 5-fluorouracil; CD-DST, collagen gel droplet-embedded culture drug sensitivity test.

CD-DST was performed in 189 out of the 246 patients (76.8%) after obtaining written informed consent according to our institutional guidelines during the study period. CD-DST was not performed in the remaining 57 patients due to various reasons, which included advanced age (≥80 years), patient refusal and underestimation of disease stage preoperatively. Out of the 189 patients, 151 patients (79.9%) underwent successful CD-DST. Failure of CD-DST in the remaining 38 patients was due to an insufficient number of viable tumor cells (<1×10^5^ in the initial assay) in 4 cases, incomplete growth of tumor cells during the culture period in 27 cases and bacterial contamination in 7 cases (Fig. 1).

Out of the 151 cases with successful CD-DST, 87 received 5-FU-based adjuvant chemotherapy for 6 months. These patients were divided into the high-sensitivity (high-group, 27/87, 31.0%) and low-sensitivity (low-group, 60/87, 69.0%) groups. The remaining 64 patients, who did not receive adjuvant chemotherapy, were included in the CTx(-) group, in order to verify the clinical utility of CD-DST (Fig. 1).

This study was a non-randomized retrospective study. The study protocol conformed to the ethical guidelines established by the Helsinki Declaration.

### CD-DST

5-FU tumor sensitivity was evaluated by CD-DST, performed as previously described by Kobayashi *et al* (8,14,15). Briefly, tumor tissue was excised from the primary surgical specimens. Subsequently, the specimens were washed twice with povidone iodine and twice with antibiotic solution containing 1 mg/ml piperacillin, 0.5 mg/ml kanamycin and 2.5 μg/ml amphotericin B. The specimens were then digested by dispersion collagenase enzyme and the dispersed cancer cells were incubated in a collagen gel-coated flask. Only the viable cells adhering to the collagen gel layer were then collected and added to reconstructed type I collagen solution (Cellmatrix Type CDTM; Nitta Gelatin Inc., Yao, Japan). Three drops of these mixtures were placed in each well of a 6-well multiplate. The plates were incubated in a CO_2_ incubator at 37°C for 24 h. 5-FU (1.0 μg/ml) was then added to each well and the plate was incubated for 24 h. Following removal of the medium containing 5-FU, the well was incubated with PCM-2 medium (Kurabo Industries Ltd., Osaka, Japan) for 7 days. Neutral red was added to stain colonies in the collagen gel droplets, which were then fixed in formalin.

The *in vitro* chemosensitivity of the tumor cells to the anticancer agent was expressed as a ratio of the total colony volume (T) of treated cells to that of untreated cells (C). Originally, samples with a T/C ratio of ≤50, ≥60 and 51–60% were defined as *in vitro* sensitive, resistant and borderline, respectively. However, in the present study, in accordance to the results of a previous study (16), the cut-off ratio was 60%, i.e., samples with a T/C ratio of ≤60% were considered *in vitro* sensitive.

### Patient follow-up

Cancer recurrence was investigated by chest X-ray examination, abdominal ultrasonography and/or chest-abdominal CT every 3 months for 1 year, every 6 months for the following 2 years and annually thereafter.

Treatment was selected among surgery, systemic chemotherapy, radiotherapy and microwave coagulation therapy, according to the guidelines for treatment of colorectal cancer (3), taking into consideration the informed consent of the patients.

### Statistical analysis

Statistical analyses were performed using the SPSS software program version 19 (SPSS Inc., Chicago, IL, USA). The Chi-square test and Fisher’s exact probability test were used to analyze data. Survival rates were estimated using the Kaplan-Meier method and the log-rank test was used to compare the curves. P<0.05 was considered to indicate a statistically significant difference.

## Results

### Comparison of clinicopathological characteristics among groups

In the CTx(-) group, the number of patients with stage II disease was significantly higher compared to those with stage III disease (P=0.001). However, no differences were observed in age, gender, tumor location, histological type, stage, lymphatic invasion, venous invasion and serum CEA and CA19-9 levels between patients in the high-, low- and CTx(-) groups. Furthermore, the chemotherapeutic agents used for adjuvant chemotherapy did not differ between the high- and low-groups (Table I).

### Overall survival time (OS) and recurrence-free survival time (RFS) according to the results of CD-DST

**Figure 2.**
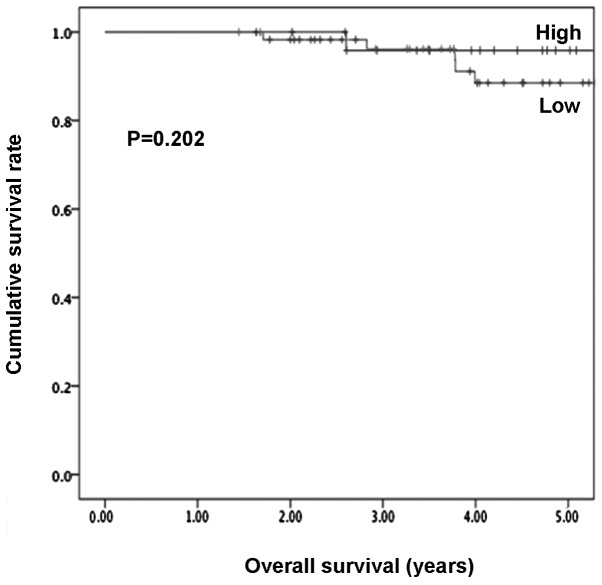
The overall survival (OS) rate was 96.3% in the high- and 86.7% in the low-group. The difference was not statistically significant (P=0.202).

**Figure 3.**
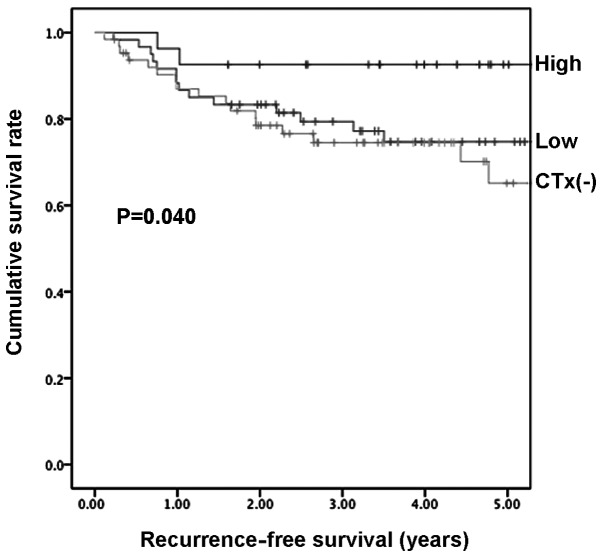
The 5-year recurrence-free survival (RFS) rate was 92.6% in the high-group, 76.7% in the low-group and 73.4% in the group that did not receive chemotherapy [CTx(-) group]. The differences between the high- and low-groups and the high- and CTx(-) groups were considered statistically significant (P=0.040).

**Figure 4.**
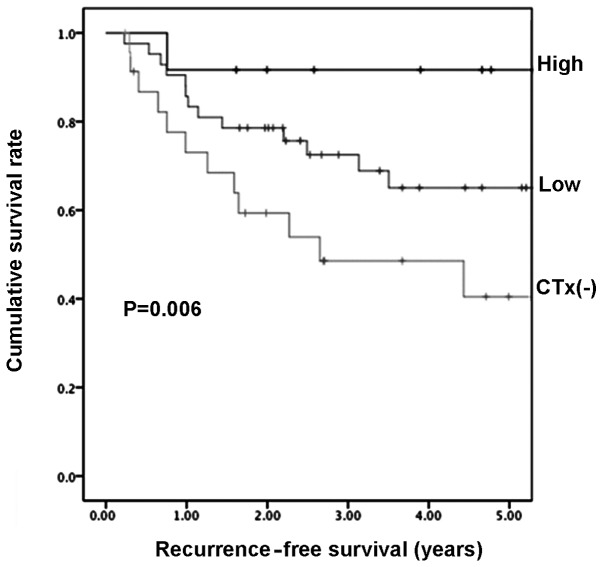
In patients with stage III cancer, the 5-year recurrence-free survival (RFS) rate was 92.3% in the high-group, 69.0% in the low-group and 50.0% in the group that did not receive chemotherapy [CTx(-)]. The differences between the high- and low-groups and the high- and CTx(-) groups were considered statistically significant (P=0.006).

The median duration from operation to follow-up was 53 months (range, 18–107 months). The 5-year OS rate was 96.3% in the high- and 86.7% in the low-group (P=0.202; Fig. 2). The 5-year RFS rate in the high-group was significantly higher compared to that in the low- and CTx(-) groups (92.6 vs. 76.7 and 73.4%, respectively, P=0.040; Fig. 3). No differences in the 5-year RFS rate were observed between the low- and the CTx(-) groups (P=0.507). In patients with stage III cancer, the 5-year RFS rate in the high-group was also significantly higher compared to that in the low- and CTx(-) groups (92.3 vs. 69.0 and 50.0%, respectively, P=0.006; Fig. 4). Furthermore, no differences in the 5-year RFS rate were observed between stage III patients in the low- and CTx(-) groups (P=0.069).

## Discussion

In Western countries, oxaliplatin (l-OHP)-based chemotherapy (FOLFOX, FLOX) remains the standard adjuvant chemotherapy for stage III colon cancer (1,2). However, Shimada *et al* (17) reported the favorable outcome (5-year OS of 87.9%) of 5-FU-based chemotherapy (5-FU + levofolinate and oral fluoropyrimidine) following curative surgery in stage III colon cancer patients in Japan. This result was more favorable compared to the results of previous studies (1,2) with l-OHP-based adjuvant chemotherapy. Therefore, administration of 5-FU-based chemotherapy for 6 months, regardless of the sensitivity of individual patients, is one of the standard adjuvant chemotherapy regimens for the management of stage III colorectal cancer in Japan. It is generally believed that adjuvant chemotherapy is unnecessary for stage II colorectal cancer patients. However, ∼25% of patients with stage II disease, such as those with penetration of the serosa, perforation, poorly differentiated histological type, or a yield of <12 lymph nodes, are considered to bear an accentuated risk of recurrence (18). Therefore, such patients are offered adjuvant chemotherapy.

It has also been reported that the incidence of grade 3 neurotoxicity was higher among patients who received l-OHP-based adjuvant chemotherapy compared to those who received 5-FU-based adjuvant chemotherapy (1,2). Furthermore, the medical costs of l-OHP-based chemotherapy were higher compared to those of 5-FU-based chemotherapy. Therefore, we aim to develop a new method that predicts the therapeutic effect of adjuvant chemotherapy and enables decision-making regarding whether patients should be administered 5-FU-based or l-OHP-based chemotherapy. Several molecular markers have been considered as satisfactory predictors of the efficacy of 5-FU-based chemotherapy (4–6), although studies on the effectiveness of *in vitro* drug sensitivity tests are limited (19).

Several *in vitro* chemosensitivity tests for malignant tumors have been developed and clinically introduced. Four tests in particular have been widely applied, since they exhibit a high success rate for primary culture, require a small number of malignant cells for testing, allow for easy quantification of anticancer effects without contamination due to fibroblasts and are cost-effective, rapid and simple. These tests include CD-DST (8,14,15), histoculture drug response assay (HDRA) (20), succinate dehydrogenase inhibition test (21) and 3-(4,5-dimethylthiazol-2-yl)-2,5-diphenyltetrazolium bromide assay (22). Among these, CD-DST and HDRA are commonly used in Japan in clinical practice. The CD-DST is used in our institute for the selection of potential individualized chemo-therapy for patients with colorectal cancer, as the HDRA usually requires high concentrations of anticancer drugs, approximately 20- to several-hundred fold of the area under the curve to the observed level *in vivo* (14,16). Furthermore, the efficacy of the CD-DST has been clinically demonstrated in several types of cancer (9–12).

In this study, the 5-year RFS in the high-group was significantly higher compared to that in the low- and CTx(-) groups, although the 5-year OS in the high-group was not significantly higher compared to that in the low-group. These results may be attributed to the limited case series and the fact that chemotherapy against colorectal cancer led to prolongation of patient survival following recurrence. Subgroup analysis demonstrated that the 5-year RFS rate among stage III patients in the high-group was significantly higher compared to those in the low-group. Furthermore, the 5-year RFS rate in the high-group in this study was comparable, if not superior, to the 5-year RFS rate of l-OHP regimens reported by previous studies (1,2). However, no differences were observed among stage II patients (data not shown). Moreover, no differences in the 5-year RFS in stage II and III patients were observed between the low- and CTx(-) groups. These results suggested that patients in the high drug sensitivity group exhibited a prolonged RFS period compared to patients with low drug sensitivity. In other words, postoperative 5-FU-based adjuvant chemotherapy may not have exerted an effect on RFS in patients who were classified as exhibiting poor drug sensitivity. These results indicate that l-OHP-based regimens may not be required in the high-group; however, more potent regimens, such as l-OHP-based regimens, may be required in the low-group patients. Although ideally the comparison should have been between high-group patients who received 5-FU-based chemotherapy and those who did not, such an analysis was not possible in the present study, due to the limited number of patients with high sensitivity that did not receive adjuvant chemotherapy.

Future studies are required to determine whether the recurrence rate in the low-group may be lowered by l-OHP-based chemotherapy, whether 5-FU sensitivity testing is able to predict the therapeutic effect of l-OHP-based chemotherapy and whether additional l-OHP sensitivity testing is required to predict the therapeutic effect of l-OHP-based chemotherapy. The limitations of this study included the limited patient sample and the fact that this was a non-randomized retrospective study.

In conclusion, we have demonstrated that CD-DST appears to be useful for the prediction of the therapeutic response to adjuvant chemotherapy in patients with stage II–III colorectal cancer. Furthermore, this technology may prove useful for decision-making with regard to whether patients should be administered 5-FU-based or l-OHP-based chemotherapy. However, this study was a small-scale, retrospective analysis conducted at a single institution. Multi-center prospective randomized trials are therefore required to gain additional insight into the subject.

## Figures and Tables

**Table I.** Comparison of the clinicopathological characteristics among the high-, low- and CTx(-) groups.

Variables	Drug sensitivity			P-value
	High (T/C<60) n=27	Low (T/C>60) n=60	CTx(-) n=64	
Age (years)				
<70	17	34	25	
≥70	10	26	39	0.051
Gender				
Male	18	34	31	
Female	9	26	33	0.380
Location				
Colon	22	41	46	
Rectum	5	19	18	0.600
Histological differentiation				
High and moderate	25	57	62	
Poor and others	2	3	2	0.920
Stage				
II	15	18	40	
III	12	42	24	0.001
Lymphatic invasion				
ly 0, 1	10	19	33	
ly 2, 3	17	41	31	0.071
Venous invasion				
v 0, 1	15	30	40	
v 2, 3	12	30	24	0.470
CEA				
<5	15	25	28	
≥5	11	31	28	0.950
CA19-9				
<36	24	41	46	
≥36	0	10	4	0.080
Regimen				
Oral fluoropyrimidine	25	47	-	
i.v. 5-FU + l-LV	2	13	-	0.100

T, total colony volume of treated cells; C, total colony volume of untreated cells; CEA, carcinoembryonic antigen; CA19-9, carbohydrate antigen 19-9; 5-FU, 5-fluorouracil; LV, leucovorin; CTx, group that did not receive chemotherapy.
